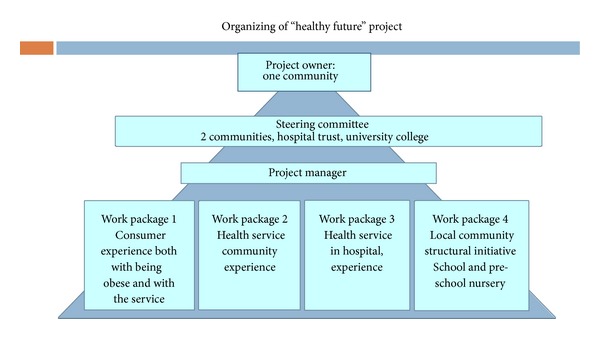# Participatory Action Research in the Implementing Process of Evidence-Based Intervention to Prevent Childhood Obesity: Project Design of the “Healthy Future” Study

**DOI:** 10.1155/2013/437206

**Published:** 2013-07-17

**Authors:** Gudbjørg Øen, Kjell Morten Stormark

**Affiliations:** ^1^Haugesund/Stord University College, Klingenbergveien 8, 5414 Stord, Norway; ^2^Regional Centre for Child and Youth Mental Health, Uni Health, Uni Research, Krinkelkroken 1, 5020 Bergen, Norway

## Abstract

*Objective*. To describe the design of the developmental project Healthy Future that aims to implement a new evidence-based program for the prevention of childhood obesity and collaboration and sharing of work between specialist and community health care professionals in parts of a county in western Norway. *Methods*. Comprehensive participatory planning and evaluation (CPPE) process as an action-oriented research approach was chosen, using mixed data sources, mixed methods, and triangulation. *Discussion*. A bottom-up approach might decrease the barriers when new evidence-based childhood prevention interventions are going to be implemented. It is crucial not only to build partnership and shared understanding, motivation, and vision, but also to consider the frames of the organizations, such as competencies, and time to carry out the interventions at the right level of health care service and adapt to the overweight children and their families needs. *Conclusion*. The developmental process of new health care programs is complex and multileveled and requires a framework to guide the process. By CPPE approach evidence-based health care practice can be delivered based on research, user knowledge, and provider knowledge in the field of childhood overweight and obesity in a certain context.

## 1. Introduction

Health care professionals in Norway are increasingly worried about the negative trend they observe in the number of overweight and obese children and adolescents. The Norwegian Child Growth Study measuring 3rd graders from a national representative sample of 127 schools, in which nine of ten children took part, demonstrated a 3% increase in ISO-BMI from 2008 to 2010, and 22% of the girls and 17% of the boys were estimated to be overweight or obese in 2010 [1]. In a more detailed study of 1774 children from Finnmark, the northernmost county in Norway, born in 1999-2000, 19% of the children (22% of the girls; 16% of the boys) were classified as overweight when they had reached the age of six [2]. Overweight negatively affects children's physical and psychosocial health [3] and compromises their future health, particularly in terms of an increased risk of obesity [4]. Thus, the developmental trend in Norway is that more and more children become more and more overweight, at an earlier and earlier age, which accords with the developmental trend in most westernized countries [5]. Even though treatment of overweight might be considered to have an easy solution (increase energy expenditure and decrease energy intake) the rate of overweight children has been steadily increasing, suggesting that the prescribed solution is an aim rather than a method [6–9]. 

Up until 1998 the public health nurses measured all children's weight and height in order to estimate growth curves for all children as part of routine health examinations at the school health service in Norway. In 1998 the Norwegian health authorities revised their recommendations for the public health nurses to recommend that only school children judged to be ill or at risk for becoming ill should be weighed. The recent obesity pandemic in children worldwide has recently led to a revision back to the original program for monitoring each child's weight and height in order to estimate ISO-BMI and growth curves, in an attempt to prevent children from becoming overweight. The Norwegian Health Directorate therefore published new guidelines for the school health service [10] where routine measurement of children's height and weight was reestablished, recommending measures to be taken at five years, 3rd and 8th grade with the goals to identify early growth aberrance, to reduce the individual negative consequences, and to make the basic for implementation and evaluation of interventions at a public health level. At the same time new national guidelines for prevention, mapping, and treatment of overweight and obesity in children and youth were published [11] to ensure proper professional service with regard to overweight problems in primary care, and to contribute to cooperation in all directions in the health care systems. The guidelines are structured by and are recommending interventions according to the level of ISO-BMI. These two sets of national guidelines are not widely implemented in the primary health care service yet.

As the monitoring of children's height and weight developments is important for identifying children at risk for obesity, it must be accompanied by effective interventions. Children or adolescents with obesity have traditionally been met by school nurse or general practitioners and told to reduce food intake and increase the physical activity. There has been no structured or systematically plan, and the responsibility for this care is experienced unclear [12]. Such a approach rely on a linear understanding that if energy intake is lower than expenditure, the weight will be reduced, but underestimate the importance of the persons feelings, thoughts, and environmental influences such as peers and parents. To help people change behavior, a psychological approach is needed. Competence in using these methods are mostly available in the mental health care, while obesity is tried solved in the field of somatic problems. Uncertainty where and by whom childhood obesity should be treated is a key question in Norway, and also if the competence exists where these children live [12].

There is limited access to evidence-based interventions for childhood overweight in Norway, and in rural areas there is also limited access to professionals with specialized training. Overweight individuals also often delay asking for help from health-care services. Many professionals in primary care are uncertain in meeting overweight children and need more knowledge of what works. The practitioners are faced with the challenge of implementing a program in their daily work and decide who in the community staff/hospital shall do what [12]. Due to limited resources and access to evidence-based programs, there is a great need for prioritizing the health care resources. This also calls for an increase in preventing efforts. It is much more demanding treating obese children than preventing overweight children from becoming obese. An increased effort on preventive measures is also called for by the fact that it is possible to identify specific risk factors for childhood obesity. One certain risk factor for becoming obese is having obese parents [13, 14], a second is when the ISO-BMI is increasing, the child starts becoming overweight [15], and parents are not aware of the situation [13]. 

The health care system seems to be faced with some challenging aspects regarding the treatment because obesity is understood with some discrepancy within the health care providers regarding treatment and preventions methods, and their differences in the understanding where and when treatment interventions should be initiated [12]. Therefore there is a great need for more research on the implementation of evidence-based treatment program for childhood obesity in primary care. From other countries it is pointed to the need for bringing pediatric weight management into practice [16], and that programs working in other countries are not always successfully implemented elsewhere [17]. Waters et al. [18] conclude that childhood obesity prevention research must move towards identifying how effective intervention components can be embedded within health, education, and care systems and achieve long-term sustainable impacts. According to Klesges et al. [19], enhanced reporting of relevant and pragmatic information in behavioral investigations of childhood obesity interventions is needed in order to improve the ability to evaluate the applicability of results to practice implementation and that such evidence would improve translation of research to practice, provide additional explanation for variability in intervention outcomes, and provide insights into successful adaptations of interventions to local conditions. The aim of this paper is to present the process of and theoretical consideration for implementing an evidence-based treatment program for overweight children in Norway, using a “bottom-up approach.” 

The increase in the number of obese children in Norway requires early interventions, particularly targeting children with overweight to prevent them from becoming obese. A multicase study from the childhood obesity field in Norway reported a great need for developing both preventive and treatment interventions that were evidence based. It was also recognized differences in the providers understanding with regard to the obesity situation as well as what kind of treatment should be chosen [12]. These findings have led us to take steps to coordinate a project with the aim to promote the implementing process of new national guidelines and based on the best evidence available achieve shared understanding among the providers in the field. After more than one year of preparations the project “Healthy Future-prevention of childhood obesity” (HF project) was established. 

Evidence-based (EB) practice requires that the professionals make decisions based on the systematic gathering of evidence drawn from research, experience, and advice of clinicians working with the patients along with the patient's input, desires, and needs [12]. Unfortunately, it is not always the case that production of “evidence” means that practitioners integrate it into their everyday practice, and often practice lags behind what is known to be current best practice [20]. Steine [21] stated that important changes in the health services seldom occur solely from a top-down approach. During implementing health care interventions we have to consider both what kind of knowledge to be implemented and how knowledge is facilitated in the context where it will be used [22]. Successful implementation is the function of knowledge, context, and facilitation according to the framework “Promoting action on Research Implementation in Health Care” (PARiHS) [23]. Research from implementing frameworks is published from a range of fields [24–31]. The Child System and Treatment Enhancement Projects (ChildSTEPs) were launched to help bridge the science-practice gap in children's mental health service [32, 33]. In this it was recognized that community mental health services were delivered through complex organizational systems, and that success of such systems is likely to be affected by several macrolevel factors that include official regulations, funding, and collaborative agreements among related service systems [34]. Translation of research findings and knowledge and making decision about its use in daily practice is challenging for health professionals [35–37]. As a response to this participatory action research (PAR) could be a methodological strategy to be used. According to research, PAR is a method that increases the possibilities for a better transformation to specific situations, facilitates the implementation of new knowledge, and in this way fills the gap between theory and practice [38–42]. Schultz et al. [43] underlines the need for a strong platform and more strategic systems approach in future obesity prevention research. Encouraging community engagement in formulating research agendas and promoting ownership of health solutions will be a key to improving obesity risk factors [44]. Community-based participatory research (C-BPR) approach is recommended in the field of obesity prevention [44–46], and others have found this to create the opportunity for partners to train together, build capacity, and increase cohesion, and develop relationships and trust [47].

## 2. The “Healthy Future” Study

### 2.1. Evidence for Changing Behavior in Childhood Overweight

During the last decades there has been an increased focus on and drive for quality improvement and demand to ensure that the delivery of health care is evidence-based and clinically effective, so also in interventions used for overweight children. Within the HF project the team needed to decide what and how they will use the current research evidence. In the latest Cochrane review of childhood obesity cognitive behavior therapy (CBT) is acknowledged as the treatment of choice. “While there is limited quality data to recommend one treatment program to be favoured over another, this review shows that combined behavioural lifestyle interventions compared to standard care or self-help can produce a significant and clinically meaningful reduction in overweight in children and adolescents” (Oude Luttikhuis et al., 2009:2) [48]. The behavioral economics model of obesity has been found to provide a particularly good framework for overcoming resistance to change in overweight children and adolescents [49] and was also associated with good long-term outcomes [50, 51]. In Iceland the Epstein model has been adapted and tested by Gunnarsdottir [52] and was found to be acceptable and effective in twenty children with overweight or obesity in a diverse sample. However, the Norwegian national guidelines did not recommend a special treatment program to be preferred and used but reported diverse evidence levels from A–D according to each initiative mentioned. In the list of recommendations we find that early onset and long-lasting treatment, multiprofessional cooperation, reducing sugar, salt, and fat are labeled evidence level B. Even in the guidelines it is stated that the health care professionals should know conventional recommendations for weight reduction in obesity treatment (page 54), and cognitive behavioral treatment (CBT) is discussed (page 55), CBT is not mentioned in the list of recommendations neither for overweight nor for obesity (page 10–12). Reading the guidelines the somatic side of obesity and its treatment are well described and emphasized, but research shows that psychological treatment methods added in the treatment plan are the most recommended and shows the best effect [48, 53].

Early onset and long-lasting treatment are highlighted and recommended [54], as well as the whole family is active in the treatment. According to Golley et al. [55] and Magarey et al. [56] parent skill training increases the efficacy of pediatric obesity interventions, and Kitzman-Ulrich et al. [57] concluded that parent training in general behavior management was associated with significantly better outcomes in family-based interventions.

The recommendations of expert panels (the US Center for Disease Control and Prevention, the American Medical Association, the American Academy of Pediatrics) and guidelines developed by the National Association of Pediatric Nurse Practitioners encourage a shift from a traditional model of counseling (by telling people what they should do) to a collaborative, family-centered model that includes the use of motivational interviewing (MI), in which the nurse and family jointly formulate a plan of care that is consistent with the family's values and priorities [58]. In the Norwegian guidelines for childhood obesity [11] MI is underlined as a tool to be used in encounters during prevention and treatment, as well as MI is described and recommended in most of the official guidelines with regard to public health initiatives in Norway. Motivational interviewing (MI) is a communicative technique that is highlighted as a method to help people change behavior with a large evidence base comprising more than 200 randomized clinical trials has emerged and is showing positive effects [59]. MI seems also to be a promising tool used in childhood obesity [60, 61]. The service to the overweight child and the effect of the treatment program will largely depend on the implementing quality. To facilitate the implementation process we used a bottom-up approach that could promote the institutionalization of our targeted interventions.

### 2.2. Aim and Objectives

The primary aim of this project is to prevent childhood obesity. 

The project intends to

1. map and define the need for initiatives aimed at families affected by obesity and risk for it,
2. map resources and barriers within the health service (personnel, expertise, competency, attitudes, and treatment/management),
3. establish a new comprehensive model for prevention, management, and followup and a model for collaboration and sharing of work between specialist health-care services and the community,
4. implement and evaluate the new model.

*Secondary aims.*

The project intends to

1. understand what intervention children, adolescents, and their families with obesity/overweight problems want from health care personnel,
2. achieve greater confidence from public health nurses and doctors in terms of raising the subject of/talking about obesity at an early stage in its development,
3. develop evidence-based methods/materials for prevention, treatment, followup and health promotion for use in health care,
4. improve provision of evidence-based practice in specialist's and community-based health care services to families with overweight and obesity,
5. bridge the gap between the expectations from the target group and what the service can provide.

This paper describes the design of the project “Healthy Future-prevention of childhood obesity” (HF-project), process of establishing network and venue to build motivation to carry out new health care service to the group of overweight and obese children and their families in one region in Norway.

One example of research question for 1st phase:

*What interventions do health care professionals offer to obese children and their family, which resources and barriers are pointed to, and what expectations with regard to childhood obesity interventions do health care providers recommend for the future? *

### 2.3. Study Setting

The Healthy Future study is a comprehensive locally developed project, with diverse partners: two local hospitals within the same hospital trust, and two different municipalities, a university college and The User Association for Obesity. The query to the hospitals went to the pediatric- and the child psychiatric units and to the child- and adolescent health prevention service in the communities. The period of recruiting partners and strengthening motivation for the project lasted more than one year, mainly because there was no economical support for the project at that time. The prolonged process caused turnover in individuals and decreased the number of communities that participate in the project. The project is carried out in the southwestern part of Norway. 

#### 2.3.1. Participants

The Healthy Future study group comprises a total of 14 professionals who all are engaged in obesity prevention work. The professionals represent two municipalities in the county which are the core competence in helping overweight children and their families. The group comprises child physiotherapists and public health nurses (from the municipalities) and pediatricians, family therapists (from the hospital), nurses, and researchers (from the University College). In addition a social worker is a representative from the Local Association for Obesity in the group. (Figure 1, work package 2).

#### 2.3.2. Organization of the Project

One municipality is defined as the project owner, which includes the administrative responsibility for running the project. One of us (GØ) acts as a manager, and the project was led by a steering committee from the Hospital Trust, both the municipalities, and the University College. The work to be done through each phase was divided into four work packages (WP): WP 1: consumer experience both with being obese and with the service, and recommendations for the future, WP 2: the experience and recommendations from the communities health providers, WP 3: the experience and recommendations from the hospital obesity treatment providers, WP 4: the experiences and recommendations from providers working in the communities (Figure 1).

Within the HF-project's WP 1, WP 2, WP 3, and WP 4, respectively, 3, 3, 2, and 1 research studies are planned or ongoing as master- and ph-d-projects with diverse additional scientific partners but will not be described in this paper. 

### 2.4. Design and Methods

#### 2.4.1. Checkland's Soft System

The project was initiated by us as a result of the multicase-study [12] by inviting partners: two hospitals and six surrounding communities, and asking if their professionals could support the process of implementing EB interventions in the field of childhood obesity. All fourteen professionals attended the first meeting, where interventions for childhood obesity were raised and discussed guided by Checkland's “Soft System” theoretical framework [62]. This model, from a qualitative methodological perspective, is a framework for changing clinical practice, as a PAR design. The model consists of seven stages: (1) assessment of the situation or practical problem and description of this problem, (2) identification of the systems involved, (3) desirable modeling of the systems, (4) comparison of the desirable model and the problematic situation, (5) establishment of a concept of what is desirable and what is possible, (6) implementation of the proposed activities and observations, and (7) reflecting about the change. Two meetings discussing the situation and the review process of written proposal resulted in financial support from the Norwegian Health directorate. After fulfilling the phases from 1 to 6, to strengthen the structure of the implementation phase, the PARiHS framework acted as a theoretical framework for the project. Successful research implementation was described as a function of a dynamic, simultaneous relationship among evidence, context, and facilitation [63]. These three key elements are described in more detail, where evidence is characterized by research evidence, clinical experience, patient experience, and local data and information; context by culture, leadership, and evaluation; and facilitation by purpose, role, and attributes, derived from the research, practice development and quality improvement work by Rycroft-Malone [63] that other researches have supported [64]. 

#### 2.4.2. Comprehensive Participatory Planning and Evaluation Process

Participatory action research (PAR) was chosen as a strategy in HF project because the aim was two-folded; to create both knowledge and new practice. According to Greenwood and Levin [65] Action Research (AR) is social research carried out by a team encompassing a professional action researcher and members of an organization or community seeking to improve their situations. AR promotes broad participation in the research process and supports action leading to a more just or satisfying situation for the stakeholders [65]. Many models and newly created frameworks are built on AR as a research practice with a social change agenda.

The comprehensive participatory planning and evaluation (CPPE) process is an action oriented approach designed to guide project planning and evaluation in communities during five steps including (1) problem assessments, (2) identification and selection of interventions, (3) planning, (4) intervention proposal development, and (5) monitoring and evaluation of the results [66], partly concurrent with the Checkland's Soft Systems, and provided for a context of complex systems as communities. A system approach underlies AR in all its manifestations. Social systems are not mere structures but are processes in continuous motion interlinked and intertwined in the individual's social structures and the larger ecology of systems into complex interacting macrosystems [65]. To build motivation for EBP it is crucial to involve each partner's participation during the process. Optimally, the practitioner has experience-based and well-considered knowledge about the practice field [67] and can offer input in important situations, whilst the researcher possesses theoretical knowledge and can offer input from these perspectives. 

#### 2.4.3. Mixed Research Methods

One way of using multiple research approaches to study the implementing process of childhood obesity interventions is through the application of mixed methods research that integrates the collection and analysis of both quantitative numeric data and qualitative narrative data. By combining these two approaches within mixed methods research designs, researches can maximize the strengths of each approach while making up for the weaknesses of the approaches, develop more complete and complementary understandings, increase validity of results, use one form to build on the results of the other, or examine contextualized understandings, multilevel perspectives, and cultural influences [68–71].

According to Creswell and Plano Clark [72] a definition of mixed methods is useful for differentiating the many perspectives people bring to defining mixed methods:

*Mixed methods research is a research design with philosophical assumptions as well as methods of inquiry. As a methodology, it involves philosophical assumptions that guide the direction of the collection and analysis and mixture of qualitative and quantitative approaches in many phases of the research process. As a method, it focuses on collecting, analyzing, and mixing both quantitative and qualitative data in a single study or series of studies. Its central premise is that the use of quantitative and qualitative approaches in combination provides a better understanding of research problems than either approach alone. (p. 5)*

Mixing refers, according to Creswell and Plano Clark [68], to the researchers determining when and how to integrate or combine the data sets. Mixing might occur at any of the four major steps in a research process: during interpretation, data analyzes, data collection, and/or during the research design process. Of the six designs from Creswell and Plano Clark [68] we chose the multiphase design as fitting the program development in HF project and its evaluation. The multiphase design occurs when the researcher combines both sequential and concurrent design elements over a period of time within a program of the study dealing with an overall programmatic objective. This design is useful when a complex approach is required to achieve multiple study objectives over time [68].

Action researchers accept a wide range of research techniques: surveys, statistical analysis, interview, focus groups, ethnographies, and life histories are all acceptable if the reason for deploying them has been agreed on by the AR collaborators and if they are used in a way that does not oppress the participants [65]. 

The number of research studies in the HF project has been growing through the working process during the first phase, and in WP1 we will use both qualitative methods as focus groups with adolescents and individual interviews with parents as well as quantitative studies using surveys to prescholars and their parents. In WP2 and 3 we will use focus groups along with survey targeting of participants from both the specialized and the community health services. In WP4 document analyses and interviews will be used. To evaluate the project we will use both process evaluation and a broad specter of methods to evaluate the effectiveness of the new interventions targeting both the providers and users. Pre- and postdata will inform the effectiveness, using different intervention groups and controls. 

### 2.5. Data Collection

The project duration will be approximately 3 years after the protocol was accepted and became financially supported in December 2011. The project follows in line with the CPPE process [66]. 

Phase 1: problem assessments and phase 2: identification and selection of interventions lasted one year. In addition to the focus groups with providers, the data originated from discussions in the team meetings, knowledge transfer through two conferences and reviewing the summaries from each of these events, and evaluation of the conferences. The conferences were organized, and theme was chosen based on updated research findings in the field of childhood obesity and what providers expressed they need knowledge of to improve the health care service to this target group. One critical milestone is to agree on what interventions should be implemented. 

Phase 3: planning and phase 4: intervention proposal development will last for eight months. In this phase the focus will be on the qualities of the context according to PARiHS framework. This involves negotiation and developing a shared understanding about the benefits, disadvantages, risks, and advantages of the new over the old during a dialectical process that requires careful management and choreography and one that is not done in isolation; in other words, it is a team effort [23].

Phase 5: monitoring and evaluation of the results will last for one year and four months. This phase, as the implementation stage by carrying out the interventions and the evaluation, represents one important empirical part of the HF project. In this phase useful data would be pre-post evaluation tests, both summary scores for evidence and context, narrative summary, and evaluation of facilitation approach, according to PARiHS [23]. 

### 2.6. Data Analysis

The data analysis varies depending on the method used in each study. Of the qualitative analyses, in the focus group discussions the content analysis is used [73]. Transcribed texts are analyzed in five steps. In the first step the interviews are read through and listened to several times. In the second step meaning units related to the aim are identified. In the third step the meaning units are condensed and labeled and finally coded on the basis of their content. Based on the codes, subcategories and categories are developed in the fourth step. In the fifth step the categories were carefully discussed until main categories are identified. The less-complex-content analysis method by Malterud is used in some of the studies [74] inspired by Giorgi [75]. This procedure consists of the following steps: (1) total impression—from chaos to themes; (2) identifying and sorting meaning units—from themes to codes; (3) condensation—from code to meaning; (4) synthesizing—from condensation to descriptions and concepts [76].

The quantitative data will be analyzed by appropriate statistical technique, according to the research questions and hypothesis. 

To ensure the integration of mixed data, a triangulation process can be used in the meaning to describe a process of studying a problem using different methods to gain a more complete picture [77]. Triangulation techniques require researchers to list the findings from each component of a study on the same page and consider where findings from each method agree (convergence), offer complementary information on the same issue (complementary), or appear to contradict each order (discrepancy or dissonance) [78–80]. For example, interviews might be carried out with a sample of survey respondents, creating a subset of cases (individuals or groups) for which there is both a completed questionnaire and a transcript [81]. In the HF project a mixed method matrix could be used to study the relation between findings from research, interviews, and surveys according to the example of O'Cathain et al. [77, 82].

### 2.7. Ethical Considerations

The Declaration of Helsinki [83] provided the guidelines for the whole project. The multiple studies have been presented to the Regional Committee for Medical and Health Research Ethics (REC). No evaluation was necessary. The Norwegian Social Science Data Services (NSD) approved the project (number 29263).

## 3. Discussion 

Attempts to promote change may fail if the innovator adopts an unstructured approach [84]. The HF is complex project and a process of different levels: both developing new interventions and developing new implementation strategies, both designed for community health care services, as well as strategies for collaboration and sharing of work between specialist and community health-care services. These developments are built on both qualitative and quantitative data, and from the three kinds of sources: research findings, user information/data, expert information/data, and decision making and using the most suitable techniques in a mixed method design at each stage in the CB-PA process. The “Bottom-up perspective” is selected in HF project in order to decrease the barriers when new evidence-based interventions are going to be implemented. The initial phases of CB-PR, forming a partnership, assessing, and strengthening the community dynamics, are considered to be most critical [85, 86]. It is crucial not only to build shared understanding, motivation, and vision, but also to consider the frames of the organizations, such as competencies, and time to carry out the interventions at the right level of health care service and adapt to the obese child and their families needs, in a way comparison of the desirable model and the problematic situation [62]. The bottom-up perspective will hopefully be time consuming in the planning/developing process, but the intervention will be more sustainable and more easily institutionalized. As system support we offered conferences as part of the motivation process. This was evaluated to be useful and relevant for a wide range of professionals, and the conferences also acted as a meeting point for interested professionals to a wider region, which in turn will promote discussions in the field lacking health care services. In addition it is anticipated that the organizations must prioritize formal education in health care prevention and treatment for providers to be involved. 

It is expected that “The Healthy Future” project will present knowledge about how to develop coordinated services for the obese child and his/her family and clarify the responsibilities and duties for hospital units and the communities, as well as the duties of the community as a whole, to promote health and that the study design and process will increase the ability to implement new practice both in hospital and community settings. 

### 3.1. Limitations

The following are considered possible limitations for this project: (1) initiative of the project was taken of a researcher situated away from the daily concerns of the healthcare professionals. (2) The HF project took a long time to be established, and since its origin several enthusiastic providers that acted as door openers have changed work positions, which in turn require additional time and energy to build motivation in new personnel. (3) The design as a whole with the qualitative approach adapted to a special region area and results cannot be generalized but merely support knowledge to similar contexts and situations.

## 4. Conclusion 

In conclusion, using a community-based participatory research approach in the Healthy Future Study shows an example of a method to be used in implementing childhood overweight and obesity preventive and treatment interventions in one certain region. The developmental process of new health care programs is complex and multileveled and requires a framework to guide the process. Mixed methods approach is demanding, but can be useful if the most appropriate triangulation technique is used during the different project steps. By this approach evidence-based practice can be delivered based on research, user knowledge, and provider knowledge in the field of childhood overweight and obesity in a certain context.

## Figures and Tables

**Figure 1:**